# Effect of a Triad of Norepinephrine, Tranexamic Acid, and Endo-Cutter Staplers on the Reduction of Blood Loss and Operative Duration in Patients Undergoing Open Radical Cystectomy

**DOI:** 10.7759/cureus.84569

**Published:** 2025-05-21

**Authors:** Harkirat S Talwar, Sudheer Kumar Devana, Vikas Panwar, Pranav Sharma, Shrawan K Singh, Santosh Kumar

**Affiliations:** 1 Urology, Max Super Speciality Hospital, Noida, IND; 2 Urology, Postgraduate Institute of Medical Education and Research, Chandigarh, IND; 3 Urology, All India Institute of Medical Sciences, Rishikesh, IND

**Keywords:** blood loss, norepinephrine, open radical cystectomy, tranexamic acid, vascular staplers

## Abstract

Objective: To evaluate the effect of norepinephrine, tranexamic acid, and endo-cutter staplers on the reduction of blood loss and operative duration in patients undergoing open radical cystectomy (ORC).

Methods: We conducted a prospective case-control study involving 50 patients who underwent ORC. The first group consisted of 25 patients (cases) who received norepinephrine and tranexamic acid-soaked mops, along with endo-cutter staplers for ligating the vascular pedicles during the surgery. The second group comprised 25 patients (controls) who did not receive these interventions. Intraoperative blood loss, blood transfusions, operative time, and perioperative outcomes were evaluated in both groups.

Results: Clinical and tumor characteristics were comparable between the two groups. A significant reduction in mean intraoperative blood loss was observed in the intervention group (282.00±121.74 mL) compared to the control group (502.00±184.54 mL; p=0.000). A significant reduction was also seen in the mean number of blood units transfused (0.24±0.52 vs. 1.32±0.94; p=0.000), the mean fall in hematocrit (2.56±1.09 vs. 7.56±3.27; p=0.003), and the mean intraoperative time (2.89±0.604 hours vs. 5.36±1.295 hours; p=0.000). The mean postoperative hospital stay was significantly shorter in the intervention group (10.16±2.39 days vs. 18.32±8.38 days; p=0.000). No intraoperative complications related to the drugs were noted.

Conclusion: The use of topical norepinephrine, tranexamic acid, and endo-cutter staplers results in a significant reduction in intraoperative blood loss, blood transfusion rates, and operative duration in patients with urothelial bladder cancer undergoing ORC.

## Introduction

Carcinoma of the urinary bladder is one of the most common malignancies of the urinary tract. It ranks ninth among the most commonly diagnosed cancers worldwide [1]. In the Indian context, it is the ninth most common cancer, accounting for 3.9% of all cancer cases [2]. Surgical management of bladder carcinoma includes transurethral resection of the bladder tumor for the diagnosis and treatment of non-muscle invasive neoplasms and radical cystectomy (RC) with pelvic lymphadenectomy for invasive cancers. Achieving safe long-term oncologic outcomes, reasonable functional results, and preserving the quality of life are the primary goals of contemporary surgical approaches in patients undergoing RC for bladder cancer.

RC is a technically challenging procedure, and goals such as speedy postoperative recovery, short hospital stays, and a reduction in morbidity and mortality are difficult to achieve. Although mortality rates have significantly decreased over the years, morbidity remains static, ranging from 11% to 68% [3]. Open radical cystectomy (ORC) is associated with substantial blood loss and a high incidence of perioperative blood transfusions. On average, a patient loses between 560 mL and 3000 mL of blood during ORC [4,5]. Blood transfusions themselves come with potential complications, further increasing the total hospital costs for RC. Therefore, strategies to reduce blood loss and the need for transfusions are necessary.

Meticulous surgical techniques, including efficient dorsal venous plexus ligation, careful hemostasis, use of epidural anesthesia, and intraoperative fluid restriction, can help reduce blood loss. Additionally, recent technical advancements such as the bipolar device (LigaSure) and the harmonic scalpel have become valuable tools in the surgeon's repertoire.

A small, single-center randomized trial concluded that the use of a stapling apparatus during ORC can reduce blood loss and significantly decrease the need for transfusions [6]. Moreover, anti-fibrinolytic agents such as tranexamic acid have been thoroughly studied in various surgical procedures to reduce blood loss and the need for allogeneic blood transfusions. Tranexamic acid, a synthetic derivative of the amino acid lysine, inhibits the activation of plasminogen to plasmin by competitive inhibition, thus preventing the degradation of fibrin clot by plasmin. A recent systematic review by Henry et al. showed that the use of anti-fibrinolytics in elective surgeries reduced blood transfusion rates by 33%, decreased the volume of blood needed per transfusion by one unit, and halved the need for subsequent surgeries to control bleeding [7].

Local application of norepinephrine induces vasoconstriction. Additionally, norepinephrine is a platelet-stimulating agent that promotes platelet aggregation through α2-adrenoceptors [8]. These actions may explain the efficacy of norepinephrine in reducing perioperative blood loss through peripheral vasoconstriction, as well as postoperative blood loss through its hemostatic effect.

In the present study, we evaluated the efficacy of using norepinephrine and tranexamic acid-soaked gauze pieces, along with endo-cutter staplers during RC, to reduce blood loss and consequently minimize the need for blood transfusions. This approach also aimed to reduce the exposure of patients to the complications associated with transfusions. Furthermore, we studied the impact of these interventions on intraoperative hemodynamics and postoperative recovery.

## Materials and methods

The primary aim of this study was to evaluate the efficacy of norepinephrine- and tranexamic acid-soaked gauze pieces, as well as endo-cutter staplers, in reducing intraoperative blood loss in patients undergoing ORC, by assessing the reduction in hemoglobin/hematocrit levels and the need for blood transfusions. Secondary objectives were to ascertain the safety of drugs used both in terms of intraoperative hemodynamics and postoperative adverse effects and also to study the postoperative surgical outcome in terms of postoperative complications and duration of hospital stay.

It is a single-center, prospective case-control study conducted over a span of 18 months (August 2017 to January 2019). All patients (any age and sex) undergoing ORC willing to give informed consent were included in the study. Patients with bleeding and coagulation disorders, metastatic disease, cardiac and respiratory disorders rendering the patient unfit for surgery, acquired defective color vision, subarachnoid hemorrhage, chronic kidney disease (serum creatinine >2.0), or hypersensitivity to tranexamic acid were excluded from the study.

A total of 50 patients who met the inclusion criteria were recruited for the study. This was a non-randomized case-control study with a 1:1 ratio, where cases and controls were selected alternately. Full informed consent was obtained from each eligible patient prior to enrollment. Demographic and clinical data were recorded at the time of recruitment. This included a complete medical history and physical examination, along with relevant laboratory and radiologic investigations. All patients were scheduled to undergo ORC with urinary diversion.

The participants were divided into two groups: cases and controls. In the first group of 25 patients (cases), norepinephrine- and tranexamic acid-soaked gauze pieces, along with endo-cutter staplers, were used during the procedure. In the second group of 25 patients (controls), these interventions, namely norepinephrine, tranexamic acid, and endo-cutter staplers, were not used.

During ORC, at the level of the bladder’s vascular pedicle, the ureters were divided, and the lateral vascular pedicles, including the superior, middle, and inferior vesical arteries and veins, were identified and managed using endovascular staplers in the intervention group. In the control group, traditional vessel ligation techniques were used. Additionally, throughout the procedure in the intervention group, four to five gauze pieces soaked in norepinephrine (2 mg in 1 L of normal saline) and tranexamic acid (1 g in 1 L of normal saline) were utilized.

Intraoperative blood loss was estimated based on the number of blood-soaked materials: one gauze piece was considered equivalent to approximately 10 mL of blood, and one sponge to 50 mL. This was added to the volume collected in the suction machine to calculate total intraoperative blood loss. The effects of the aforementioned drugs on intraoperative hemodynamics were also evaluated through hourly measurements of blood pressure and heart rate, recorded by the anesthesia team.

All patients underwent bilateral extended pelvic lymph node dissection. Urinary diversion was performed in the form of an ileal conduit with Bricker-type uretero-ileal anastomosis in all cases.

Thus, the evaluation of blood loss during ORC was carried out by measuring the mean intraoperative blood loss, the mean fall in hemoglobin and hematocrit levels at 36 hours postoperatively compared to 24 hours preoperatively, and the mean number of perioperative blood transfusions (including both intraoperative and postoperative transfusions). The safety of the drugs used, namely norepinephrine and tranexamic acid, was assessed by monitoring intraoperative changes in blood pressure and heart rate, as well as by observing any postoperative adverse effects. Postoperative outcomes of the surgery were evaluated in terms of complications and the duration of hospital stay.

Continuous data such as age, operative time, tumor size, amount of blood loss, hemoglobin, hematocrit, number of blood units transfused, and duration of hospital stay were presented as mean ± standard deviation, and comparisons were made using the unpaired t-test. Discrete categorical data such as sex, presenting complaints, comorbidities, smoking status, preoperative hydronephrosis, tumor size and location, lymph node status, extravesical extension, tumor stage, and TNM stage were expressed as numbers and percentages (%) and compared using the chi-square test. The normality of continuous data was assessed using the Kolmogorov-Smirnov and Shapiro-Wilk tests. The homogeneity of variances was evaluated using Levene’s test. All statistical tests were two-sided, with a significance level set at α=0.05 (95% CI). Statistical analysis was performed using IBM SPSS Statistics (version 23.0, IBM Corp., Armonk, NY).

## Results

**Figure 1:**
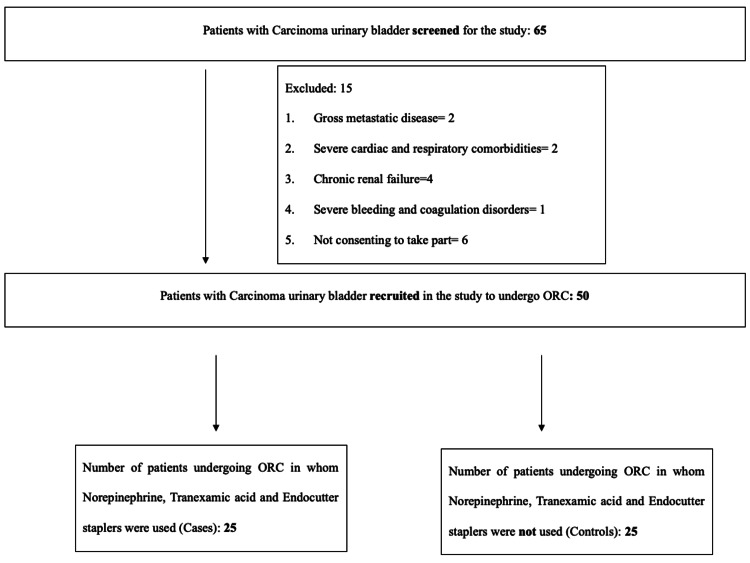
Patient enrollment into the study

A total of 65 patients were screened for the study, out of which 50 met the inclusion criteria and were recruited into the study. The participants were divided into two groups of 25 each, the intervention group and the control group (Figure 1). This is a non-randomized case-control study with a 1:1 ratio, where cases and controls were selected alternatively. The mean age of cases was 58.96 (range: 40 to 82) years and controls was 58.12 (range: 36 to 87) years (p=0.790). Of the 50 patients, 45 (90%) were males and five were females (10%) (p=0.637). The most common presenting complaint was hematuria (92%) followed by lower urinary tract symptoms (6%). The mean tumor size in cases was 3.312 cm (SD=1.89, range: 0.7-7) and in controls was 3.98 (SD=2.52, range: 1.1-10.6) (p=0.304). Table 1 shows the demographic data and tumor characteristics between both groups.

**Table 1:** Summary of the demographic data and tumor characteristics between both groups NMIBC, non-muscle-invasive bladder cancer; MIBC, muscle-invasive bladder cancer

S. No.	Variable	Cases (n=25)	Controls (n=25)	P-value
1.	Mean age (years)	58.96±9.51	58.12±12.46	0.790
2.	Sex			0.637
	Males	22 (88%)	23 (92%)	
	Females	3 (12%)	2 (8%)	
3.	Symptoms			
	Hematuria	23 (92%)	23 (92%)	1.000
	Lower urinary tract symptoms	11 (44%)	10 (40%)	0.774
	Pain	1 (4%)	2 (8%)	0.552
4.	Comorbidities			
	Diabetes mellitus	4 (16%)	5 (20%)	0.713
	Hypertension	4 (16%)	2 (8%)	0.384
5.	Exposure to tobacco	16 (64%)	14 (56%)	0.564
6.	Neoadjuvant chemotherapy	7 (28%)	7 (28%)	1.000
7.	Imaging characteristics			
	Mean tumor size (cm)	3.31±1.89	3.98±2.52	0.304
	Preoperative hydronephrosis	11 (44%)	12 (48%)	0.777
	Lymph nodes	7 (28%)	16 (64%)	0.011
	Extravesical growth	5 (20%)	9 (36%)	0.208
8.	Final staging			
	T stage			0.535
	NMIBC			
	Ta	0 (0%)	0 (0%)	
	T1	4 (16%)	6 (24%)	
	MIBC			
	T2	13 (52%)	13 (52%)	
	T2a	1 (4%)	2 (8%)	
	T2b	3 (12%)	2 (8%)	
	≥T3	1 (4%)	0 (0%)	
	T3a	1 (4%)	0 (0%)	
	T3b	1 (4%)	0 (0%)	
	T4a	1 (4%)	0 (0%)	
	T4b	0 (0%)	0 (0%)	
	N stage			0.637
	N0	23 (92%)	22 (88%)	
	≥N1	1 (4%)	3 (12%)	
	N1	0 (0%)	0 (0%)	
	N2	0 (0%)	0 (0%)	
	N3	0 (0%)	0 (0%)	
	M stage			--
	M0	25 (100%)	25 (100%)	
	M1	0 (0%)	0 (0%)	
	Pathological stage			0.466
	I	4 (16%)	6 (24%)	
	II	12 (48%)	14 (56%)	
	III	6 (24%)	2 (8%)	
	IV	3 (12%)	3 (12%)	

Twenty-three (46%) patients (11 cases and 12 controls) had hydronephrosis on preoperative imaging. Twenty-three (46%) patients (seven cases and 16 controls) had lymph nodes >1 cm on a CECT scan. A lymph node more than 1 cm in size on imaging was considered positive for the purpose of this study. Fourteen (28%) patients (five cases and nine controls) had extravesical extension on imaging. None of the patients on bone scan showed bony metastasis. The p-values for hydronephrosis, lymph nodes, and extravesical extension were 0.777, 0.011, and 0.208, respectively (Table 1).

The patients were categorized into non-muscle-invasive bladder cancer (NMIBC), which included Ta and T1 stages, and muscle-invasive bladder cancer (MIBC), which included T2 and ≥T3 stages. In total, four cases (16%) and six controls (24%) had T1 disease that was endoscopically unmanageable and, therefore, were planned for ORC. T2 disease was observed in 14 cases (56%) and 15 controls (60%), while T3 or higher disease was present in seven cases (28%) and four controls (16%) (p=0.535). The groups were further categorized based on lymph node involvement as determined by histopathology. Node-negative disease was found in 23 cases (92%) and 22 controls (88%) (p=0.637) (Table 1).

**Figure 2:**
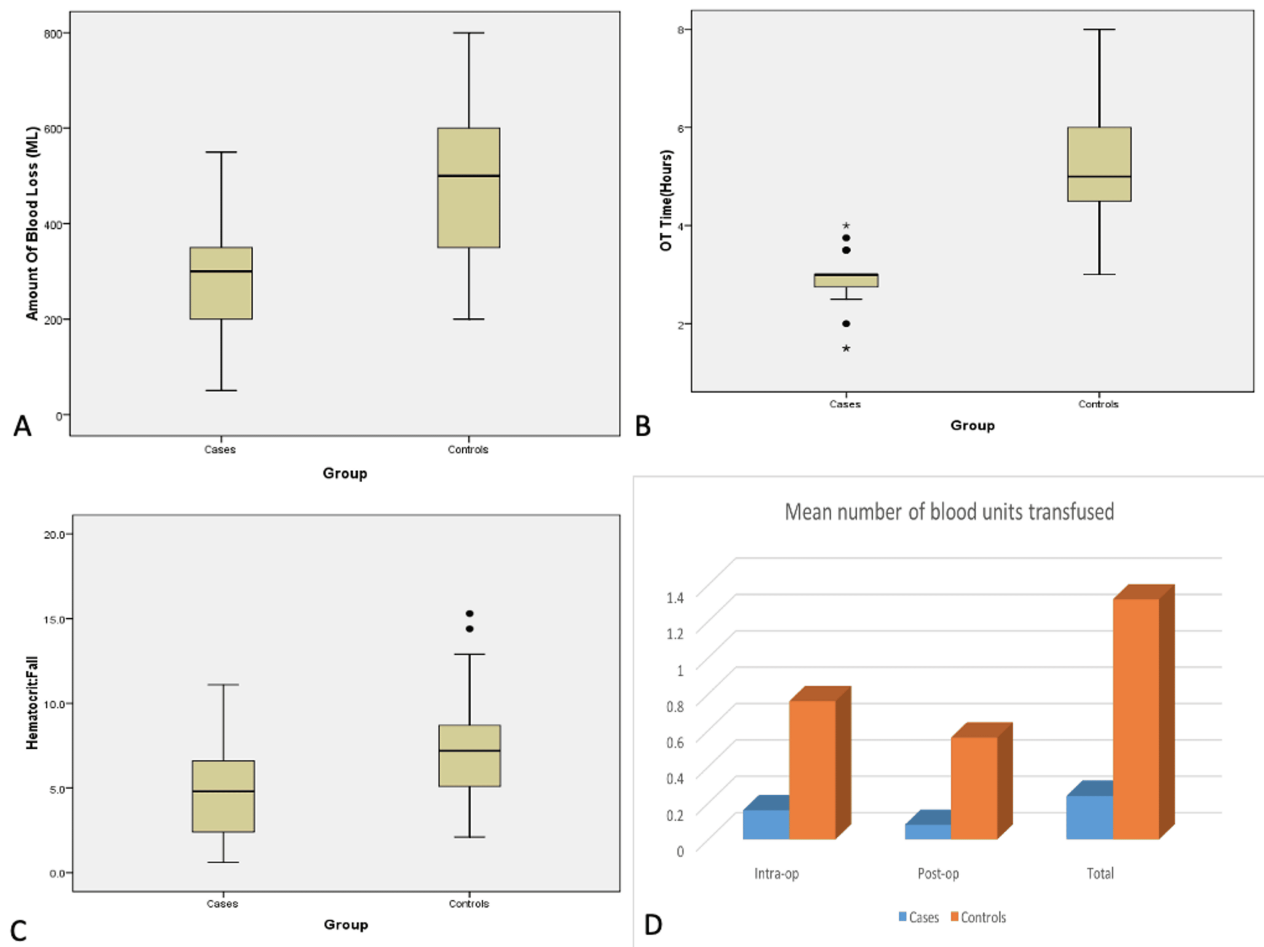
Graphical representation comparing intraoperative blood loss (a), operative time (b), fall in hematocrit (c), and number of blood units transfused (d) between the intervention and control groups

In the intervention group, the mean operative time was 2.89 hours (SD=0.604), significantly shorter than the control group, which had a mean of 5.36 hours (SD=1.295) (p=0.00). Mean intraoperative blood loss in the intervention group, where noradrenaline- and tranexamic acid-soaked sponges along with endo-cutter staplers were used, was 282.00 mL (SD=121.749), compared to 502.00 mL (SD=184.549) in the control group (p=0.00). Intraoperative hypotension, defined as systolic blood pressure (SBP) <90 mmHg, was observed in one patient in the intervention group and in two patients in the control group. The mean postoperative hemoglobin drop was 1.604 g/dL (SD=0.9719) in the intervention group and 2.560 g/dL (SD=1.0909) in the control group (p=0.002). Similarly, the mean fall in hematocrit was 4.8% (SD=2.9157) in the intervention group and 7.5% (SD=3.2772) in the control group (p=0.003). The total blood transfusion rate (intraoperative and postoperative combined) was 20% (five out of 25) in the intervention group, with a total of six units transfused (mean=0.24), compared to 80% (20 out of 25) in the control group, with a total of 33 units transfused (mean=1.32). The mean number of blood units transfused was 0.24 (SD=0.52) in the intervention group and 1.32 (SD=0.94) in the control group (p=0.00) (Table 2, Figure 2).

**Table 2:** Summary of the comparative parameters between both the groups

No.	Parameter	Group 1	Group 2	p-value
1.	Mean operative time (hours)	2.89±0.604	5.36±1.295	0.000
2.	Mean intraoperative blood loss (mL)	282.00±121.74	502.00±184.54	0.000
3.	Hemoglobin			
	Mean preoperative hemoglobin (g/dL)	11.77±1.44	11.80±1.59	0.941
	Mean hemoglobin at 36 hours post-op (g/dL)	10.16±1.36	9.24±1.42	0.023
	Mean fall in hemoglobin (g/dL)	1.60±0.97	2.56±-1.09	0.002
4.	Hematocrit			
	Mean preoperative hematocrit (%)	35.31±4.34	35.29±4.71	0.985
	Mean hematocrit at 36 hours post-op (%)	30.50±4.09	27.73±4.28	0.023
	Mean fall in hematocrit (%)	4.81±2.91	7.56±3.27	0.003
5.	Mean number of blood units transfused			
	Intraoperative	0.16±0.374	0.76±0.663	0.000
	Postoperative	0.08±0.277	0.56±0.651	0.002
	Total	0.24±0.522	1.32±0.945	0.000
6.	Mean total hospital stay (days)	18.44±7.589	30.88±7.589	0.000
	Mean postoperative hospital stay (days)	10.16±2.392	18.32±8.380	0.000
7.	Mean post-op Ryle’s tube removal (postoperative day)	3.68±1.819	6.28±4.208	0.005
8.	Mean post-op drain removal (postoperative day)	7.04±1.81	8.60±2.00	0.591
9.	Postoperative complications			
	Surgical site infections: present/absent	2 (8%)/23 (92%)	9 (36%)/14 (64%)	0.017
	Paralytic ileus: present/absent	7 (28%)/18 (72%)	12 (48%)/13 (52%)	0.145
	Mean post-op Ryle’s tube removal (postoperative day) (paralytic ileus present)	6.00±1.154	9.58±3.80	0.107
	Mean postoperative hospital stay (days) (paralytic ileus present)	11.29±1.799	24.17±8.402	0.055
	Mean post-op Ryle’s tube removal (postoperative day) (no paralytic ileus)	2.77±1.060	3.23±1.091	0.990
	Mean postoperative hospital stay (days) (no paralytic Ileus)	9.72±2.492	12.92±3.013	0.739

The mean number of postoperative hospital days was 10.16 (SD=2.392) in the intervention group and 18.32 (SD=8.380) in the control group (p=0.00). The mean postoperative day on which the Ryle’s tube (RT) was removed was 3.68 (SD=1.819) in the intervention group and 6.28 (SD=4.208) in the control group (p=0.005). The mean postoperative day of drain removal was 7.04 (SD=1.813) in the intervention group and 8.60 (SD=2.000) in the control group (p=0.591). Surgical site infection was observed in 8% (2/25) of cases and 36% (9/25) of controls (p=0.017). Postoperative paralytic ileus occurred in 28% (7/25) of cases and 48% (12/25) of controls (p=0.145) (Table 2, Figure 2).

## Discussion

Historically, RC has been associated with significant blood loss and a considerable need for transfusions. However, the reasons for this have not been clearly elucidated. This issue is compounded by the fact that many patients present with anemia due to ongoing hematuria. Recent studies [9-11] on RC have demonstrated that the mean estimated blood loss ranges from several hundred milliliters to nearly 2000 mL. The risk of substantial intraoperative blood loss has prompted numerous technical modifications aimed at reducing bleeding during ORC [10,12-14].

These modifications include surgical approaches such as laparoscopic or robotic-assisted laparoscopic cystectomy, as well as intraoperative adjuncts. The latter are further categorized into topical hemostatic agents, systemic hemostatic agents, and procedural hemostatic techniques. A review by Punjani et al. [15] examined the use of these agents and surgeon preferences, finding that approximately 88% of urologic surgeons preferred topical agents over systemic therapies.

The use of tranexamic acid as a topical agent has been explored in various surgical fields, including orthopedic, gynecological, urological, and cardiovascular surgeries. Pourfakhr et al. [16] studied local tranexamic acid use in prostatectomy and found that the mean blood loss in the intervention group was 340 mL compared to 515 mL in the control group, a reduction of nearly 34%. A systematic review and meta-analysis by Ker et al. [17] evaluated tranexamic acid’s effects on surgical bleeding, revealing a 29% reduction in blood loss and a 45% decrease in blood transfusions. Norepinephrine has also been employed as a hemostatic agent in various surgeries, including ORC. Wuethrich et al. [18] used norepinephrine as a continuous infusion during ORC and demonstrated a 33% reduction in blood loss, with median blood loss decreasing from 1200 mL to 800 mL. Blood transfusion rates also dropped by 28%. Chang et al. [6] investigated blood loss and transfusion requirements during ORC using a new stapling device compared to traditional ligation of vascular pedicles. The study reported a 30.8% reduction in blood loss (756 mL vs. 523 mL) and a 28.6% decrease in transfusions (34.3% vs. 5.7%) with the stapler group, which experienced no complications, highlighting staplers as an attractive surgical option.

In the present study, a significant reduction in mean intraoperative blood loss was observed in the intervention group (282 mL) compared to the control group (502 mL), representing a 43% reduction with the use of norepinephrine, tranexamic acid, and endo-cutter staplers. A significant decrease in the mean fall of hemoglobin and hematocrit was also noted in the intervention group.

This reduction is attributed to the vasoconstrictive and platelet-stimulating effects of norepinephrine [17] and the anti-fibrinolytic properties of tranexamic acid [19]. These effects resulted in less blood oozing during surgery, maintaining a clearer surgical field. Consequently, better visualization contributed not only to reduced blood loss but also to faster completion of the procedure. Additionally, the use of endovascular staplers [6] enabled quicker ligation of vascular pedicles, further reducing blood loss and improving hemostasis. This led to fewer blood transfusions, with significant differences in the mean number of intraoperative (0.16 vs. 0.76), postoperative (0.08 vs. 0.56), and total (0.24 vs. 1.32) blood units transfused. The intervention group also showed lower rates of intraoperative (16% vs. 64%), postoperative (8% vs. 48%), and total (20% vs. 80%) blood transfusions, reflecting the impact of the interventions on intraoperative blood loss.

The shorter mean operative times in the intervention group (2.89 vs. 5.36 hours) can be attributed to both the use of endovascular staplers for ligating pedicles and the reduced total blood loss during surgery. Endovascular staplers saved significant time by facilitating rapid dissection and ligation of each vessel in the bladder’s vascular pedicle, allowing for quicker bladder removal and faster procedure completion.

When comparing the estimated blood loss in our study to that reported in the meta-analysis by Tang et al. [20], our study demonstrated a marked reduction in intraoperative blood loss. The meta-analysis included studies comparing laparoscopic cystectomy to ORC, with no intervention in the ORC arms. Our ORC results are summarized alongside these studies in Table 3.

**Table 3:** Comparison of blood loss parameters in patients undergoing ORC in various studies ORC, open radical cystectomy

Study	Sample size (n)	Estimated blood loss (mL)	Operative​​ time (min)	Blood transfusion
Akin, 2013 [21]	15	423±79	345±112	Mean 1.0 U
Huang, 2008 [22]	108	1100±715	310±57.1	-
Guillotreau, 2009 [23]	38	923.2±532.5	334.1±93.1	Blood transfusion rate=36.7%
Khan, 2012 [24]	58	1351.9±157.2	319.8±14.1	Blood transfusion rate=58%
Wang, 2010 [25]	31	812.8±580.9	362.6±79.4	-
Present study (cases)	25	282±121.7	173.4±36.24	20%
Present study (controls)	25	502±184.5	321.6±77.7	80%

The drugs used, norepinephrine and tranexamic acid, were found to be relatively safe intraoperatively, with no significant variations in blood pressure or heart rate. No postoperative complications related to these drugs, such as visual disturbances, headaches, or musculoskeletal pain, were reported. Although previous studies involving tranexamic acid have also not reported major complications, they are limited by selection bias, as patients with a history of stroke, pulmonary embolism, or deep vein thrombosis (DVT) were typically excluded. Consequently, there remains concern among surgeons regarding the use of intravenous tranexamic acid in high-risk patients. In such cases, topical tranexamic acid serves as a safer and more suitable alternative.

**Figure 3:**
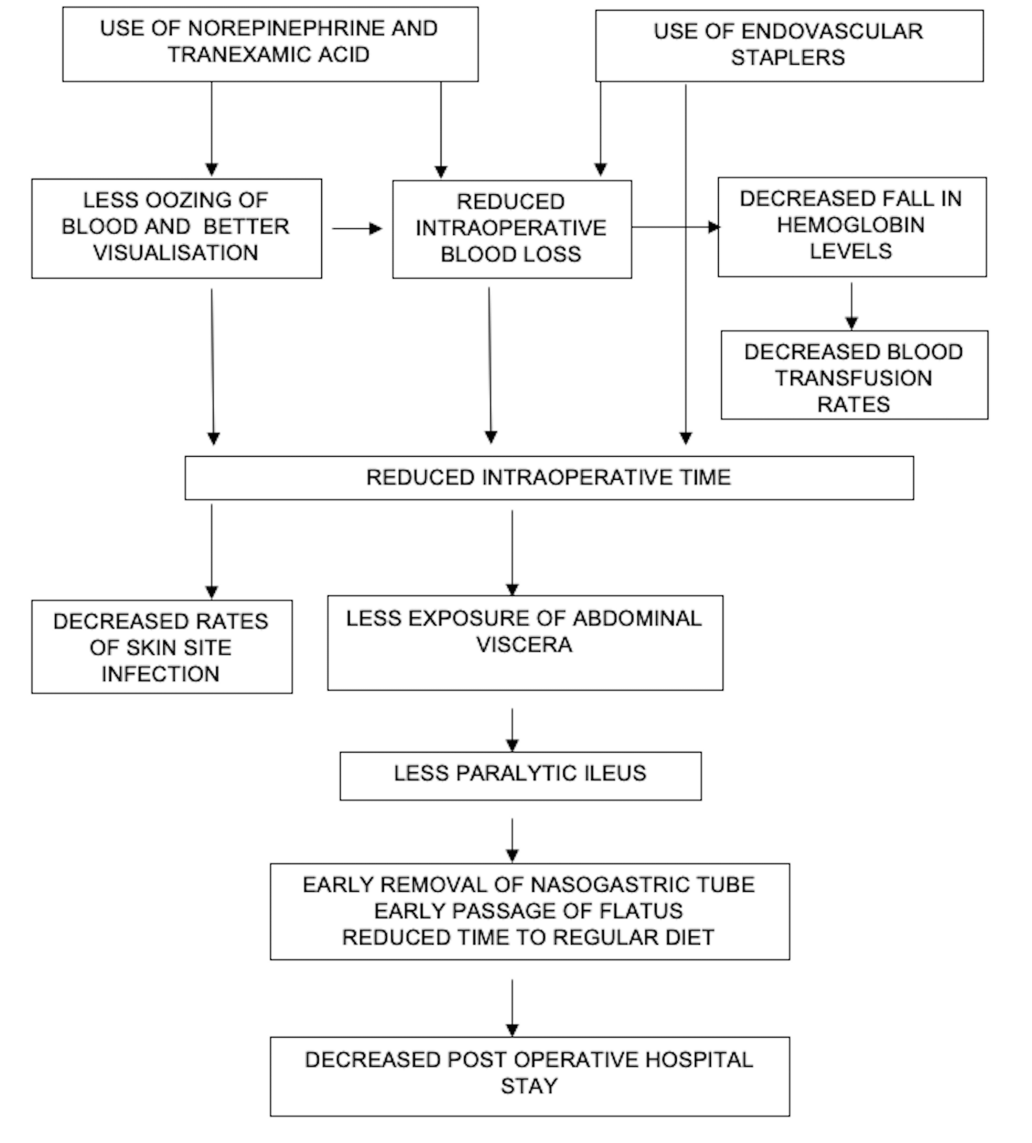
Flowchart depicting the effects of the interventions used in the study on blood loss parameters and postoperative surgical outcomes

A significant reduction in postoperative hospital stay was observed in the intervention group (10.16 vs. 18.32 days) compared to the control group. This difference was attributed to fewer postoperative blood transfusions and a lower incidence of procedure-related complications in the intervention group. The reduced intraoperative blood loss led to shorter operative times and quicker completion of the procedure, resulting in less exposure of the abdominal viscera and a reduced risk of postoperative paralytic ileus. These factors contributed to the earlier removal of Ryle’s tube, quicker resumption of oral intake, and faster return of bowel function. The shorter postoperative stay in the intervention group was thus associated with early initiation of a regular diet, earlier passage of flatus and stools, and earlier ambulation. Overall, the intervention group demonstrated faster postoperative recovery and earlier return to normal daily activities (Figure 3).

A wide range of studies support various strategies and techniques aimed at reducing intraoperative blood loss during ORC. However, most of these studies evaluate the efficacy of a single intervention. Our study is the first of its kind to examine a combination of three interventions, topical norepinephrine, topical tranexamic acid, and endo-cutter staplers, with the goal of minimizing intraoperative bleeding and the subsequent need for blood transfusions.

Several limitations of the study must be acknowledged. First, it was conducted at a single tertiary care center with a relatively small patient population. While the doses and dilutions of the drugs were standardized (2 mg norepinephrine in 1 L of normal saline and 1 g tranexamic acid in 1 L of normal saline), the number of sponges used, and therefore the total amount of drug delivered, varied between patients. Intraoperative blood loss was measured using crude methods, and no uniform criteria were established for initiating blood transfusions. Furthermore, because three interventions were used simultaneously, it is difficult to determine which intervention contributed most significantly to the observed reduction in blood loss. The cost of endo-cutter staplers also poses a limitation, as their affordability may be prohibitive for patients from lower socioeconomic backgrounds.

Another important limitation is the imbalance in lymph node positivity between the intervention and control groups. The control group had a significantly higher rate of positive lymph nodes, which likely reflects the non-randomized nature of the study. Participants were allocated alternately in a 1:1 ratio, rather than through true randomization. As a result, baseline characteristics, including oncological burden, may not have been evenly distributed. Positive lymph node status is associated with more extensive pelvic dissection, increased operative time, and greater blood loss, all of which may have biased the results against the control group and exaggerated the treatment effect observed.

This study specifically focuses on patients undergoing ORC, which remains the standard approach in settings where robotic-assisted radical cystectomy (RARC) is not financially accessible or covered by insurance. In our patient population, economic constraints often dictated the surgical method, with ORC chosen for those unable to afford robotic procedures. Although RARC is gaining popularity, particularly in high-resource settings, and is associated with reduced blood loss, fewer transfusions, shorter operative times, and quicker recoveries, our findings are intended to inform practice in resource-limited environments where ORC remains the standard of care. As such, the applicability of our results to robotic surgery is limited, and further studies are needed to evaluate whether similar outcomes are achievable in RARC cases.

## Conclusions

This study demonstrates that the use of topical norepinephrine, topical tranexamic acid, and endoscopic staplers during ORC significantly reduces intraoperative blood loss, hemoglobin and hematocrit decline, and the need for blood transfusions. These findings are particularly relevant in resource-limited settings where robotic surgery is not readily accessible. However, the non-randomized design of the study limits the ability to draw definitive conclusions. To validate these results and support broader clinical adoption, well-designed randomized controlled trials are needed. If confirmed, these interventions could improve perioperative outcomes, reduce transfusion-related complications, and provide a cost-effective standard of care for appropriately selected ORC patients.
